# Implant Apicoectomy as a Minimally Invasive Alternative to Explantation in Retrograde Peri-Implantitis: A Case Report with a 12-Month Clinical and Radiological Follow-Up

**DOI:** 10.3390/dj14060322

**Published:** 2026-05-28

**Authors:** Krystyna Kabacińska, Michał Karpiński, Patrycja Pawlik, Andżelika Warcholak-Grzeszewska, Katarzyna Barczak

**Affiliations:** 1 Department of Endodontic Surgery, Pomeranian Medical University, 70-204 Szczecin, Poland; mkarpinski@wp.pl (M.K.); patrycja.pawlik@pum.edu.pl (P.P.); andzelika.warcholak.grzeszewska@pum.edu.pl (A.W.-G.); katarzyna.barczak@pum.edu.pl (K.B.); 2 Orto Village—Centrum Ortodoncji i Implantologii, 05-552 Wola Mrokowska, Poland

**Keywords:** retrograde peri-implantitis, implant apicoectomy, implant microsurgery, CBCT, tissue preservation

## Abstract

**Background/Objectives:** Implant explantation is often considered the standard treatment for severe peri-implant inflammatory lesions, including cases with apical involvement. However, this approach is relatively invasive and aggressive, which can lead to significant loss of bone and soft tissues, potentially compromising future prosthetic rehabilitation, particularly in the esthetic zone. This study aimed to present implant apicoectomy as a conservative alternative that would allow preservation of implant stability while effectively treating localized inflammation. **Methods:** This case report presents a female patient who was diagnosed with retrograde peri-implant inflammation, and an implant apicoectomy was performed as a conservative approach to implant explantation. The procedure was carried out using a minimally invasive microsurgical technique. **Results:** The treatment resulted in complete clinical and radiographic healing, maintenance of implant osseointegration and stability, and resolution of clinical symptoms, including fistula closure. No recurrence of periapical infection was observed during the twelve-month clinical and radiological follow-up visit. **Conclusions:** Implant apicoectomy may be considered a minimally invasive and effective alternative to explantation in selected cases of retrograde peri-implantitis, preserving hard and soft-tissue architecture while maintaining implant functionality. This approach may be particularly beneficial in esthetic regions where preservation of tissue architecture is critical for optimal clinical outcomes.

## 1. Introduction

Modern implantology strives to achieve biological excellence by restoring masticatory function while preserving tissues and bones. With an increasing number of implantation procedures, more complications and failures are being reported, one of which is retrograde peri-implantitis (RPI). Retrograde peri-implantitis is a limited inflammatory condition affecting only the apical portion of the implant [1,2]. The incidence of RPI is low, ranging from 0.26% to several percent depending on the population and diagnostic criteria [1]. In cases where the implant has adequate primary and secondary stability, and there are no signs of marginal bone loss, complete implant removal can result in unnecessary bone destruction. Implant explantation, a procedure that involves creating access to the implant and its removal from the underlying bone, most often leads to extensive bone loss around the implant. In the anterior maxilla, the vestibular plate is thin and more susceptible to bone resorption, which, combined with a thin, soft-tissue biotype, poses a challenge when planning subsequent implant–prosthetic retreatment. Implant-based retreatment often requires additional vertical and horizontal bone augmentation procedures, which, in turn, prolong treatment time, increase costs, and make the final treatment result less predictable. Post-explantation bone defects may result in dimensional changes and loss of surrounding bone walls, potentially compromising future regenerative procedures [3,4,5].

With the development of modern medicine and the introduction of endodontic microsurgery in implantology, treatments have become increasingly precise and focused on preserving existing structures and minimizing surgical intervention. In a case where the inflammatory process is limited to the area surrounding the implant apex and osseointegration of the implant remains intact, resection of the implant apex allows for the elimination of the source of the infection while also maintaining the biomechanical function of the implant and tissue integrity, as well as helping maintain the appropriate esthetic effect [6,7,8].

To the best of our knowledge, reports describing implant apicoectomy as a conservative alternative to implant explantation remain limited in the literature.

This study aimed to present implant apicoectomy as a conservative microsurgical alternative to implant explantation in a patient with retrograde peri-implantitis and preserved implant stability.

## 2. Case Presentation

This case report was prepared in accordance with the CARE guidelines.

A generally healthy 40-year-old female patient presented to the dental office with chronic suppurative periapical periodontitis of tooth 21, with an active fistula near the root apex (Figure 1A).

Tooth 21 had been previously treated endodontically and restored prosthetically with a post-and-core and a metal-porcelain crown. The patient reported in her medical history that an apicoectomy had been performed twice on tooth 21, which resulted in a disruption of the root-to-crown length ratio and, consequently, excluded the possibility of further microsurgical intervention. The CBCT (Cone Beam Computed Tomography) images revealed bone thinning in the apical region of tooth 21, confirming the initial clinical diagnosis of chronic suppurative periapical periodontitis (Figure 1B).

Due to the lesion’s location in the esthetic region and the need to preserve the vestibular bone plate and soft-tissue profile, an atraumatic extraction of tooth 21 with immediate implant placement was planned. The planned treatment aimed to reduce the risk of bone resorption, preserve intact tissue architecture, and create conditions for achieving a predictable esthetic result. After completing a comprehensive examination of the patient and obtaining informed consent, the procedure was performed using local anesthesia (two ampoules of local anesthetic agent Ubistesin^®^, 3M Deutschland GmbH, Neuss, Germany). An atraumatic extraction of tooth 21 was performed to preserve the vestibular bone plate. The socket was revised, the granulation tissue was mechanically removed, and the fistula was excised using a Vista incision. The granulation tissue was then sent for histopathological examination.

The Axiom X3 (Anthogyr, Sallanches, France) system was used for implant treatment. Preoperative CBCT image analysis enabled the initial selection of the Axiom X3 (Anthogyr, Sallanches, France) implant (3.4 × 14 mm) (Figure 2).

The implant was placed using the manufacturer’s recommended protocol, and primary stabilization of >45 N·cm was achieved. Additionally, intraoperative augmentation of the peri-implant space was performed using a mixture of xenogeneic and allogenic bone substitute material in a ratio of approximately 1:3. Thickening of the surrounding soft tissue was also performed using sCTG. A connective tissue graft was harvested from the hard palate in the region of tooth 16, appropriately prepared, and de-keratinized. This graft was inserted using the tunneling method to protect and thicken the fistula site. The wound was sutured with non-resorbable sutures, a custom composite healing screw was placed on a temporary abutment (20 N·cm), and an Essex splint (Sarasota, Florida, USA) was used for esthetics (Figure 3).

Postoperative pharmacological management consisted of oral amoxicillin/clavulanic acid (1 g) twice daily for 7 days as antibiotic prophylaxis following simultaneous bone augmentation.

The sutures were removed 10 days post-op. The patient reported no problems or pain, and healing progressed uneventfully.

The patient came in for a follow-up visit 3 months later. Upon clinical examination, a fistula with purulent discharge was observed in the region of the root apex of tooth 22 (Figure 4A).

A CBCT scan was performed to exclude a periradicular infection in the region of the root apex of tooth 22. The CBCT images revealed no abnormal radiolucency in the periapical region of tooth 22; however, a thinning of approximately 4 mm in diameter was observed near the apex of the implant, which had been placed 3 months earlier (Figure 4B).

The marginal bone was preserved, and the implant had proper secondary stability, as clinically confirmed by the absence of implant mobility, the lack of pain upon percussion or pressure, and a stable marginal bone level on CBCT images. The patient was informed of her clinical situation, and the indications and contraindications were discussed. A decision was made to perform an apicoectomy of the implant, to which the patient gave her full informed consent. The original implant placement was subcrestal and was 14 mm in length. The implant length warranted an apical resection, assuming a 10–11 mm length would remain after the apicoectomy.

The microsurgical procedure for implant resection is no different from a routine apicoectomy. The procedure was performed using local anesthesia (two ampoules of local anesthetic agent Ubistesin^®^, 3M Deutschland GmbH, Germany). A horizontal incision was made in the movable gingiva surrounding the left central incisor, and a full-thickness flap was prepared. Access to the bone defect in the region of the implant apex was gained, revealing partially resorbed bone lamina and abundant periradicular granulation tissue. Granulation tissue was mechanically removed using a periodontal curette, the site was irrigated with saline, and then approximately 3–4 mm of the implant apex was removed using a diamond-coated fissure burr mounted on an accelerating contra-angle handpiece using abundant closed-circuit cooling (Figure 5A,B).

Hemostasis was achieved, and monofilament, non-resorbable sutures were placed (Figure 5C).

Postoperative CBCT confirmed complete removal of the apical fragment and adequate surgical management of the lesion (Figure 5D).

A follow-up visit three months post-implant resection revealed complete healing of the soft tissues surrounding the surgical site, and CBCT images confirmed complete healing of the osteolytic lesions surrounding the resected apex of the implant in the region of tooth 21 (Figure 6).

After qualifying the implant as a fully functional prosthetic abutment, a final zirconia-ceramic crown with a titanium Anthogyr base was placed (Figure 7).

The subsequent follow-up visit took place 1 month later, at which time clinical examination revealed no abnormalities. Esthetics and function of the prosthetic crown were preserved, the marginal periodontium was healthy, and the radiographic images confirmed complete healing of the periapical area of the resected implant in the region of tooth 21. The alveolar bone level was unchanged, and the emergence profile of the cervical area of the crown maintained a complete and satisfactory esthetic appearance. The subsequent follow-up visit took place 12 months later. Upon clinical examination, no abnormalities were observed; healing was appropriate, probing depth was less than 3 mm, and no bleeding on probing was observed, as well as no signs of suppuration or implant mobility. Esthetics and functionality of the prosthetic crown were preserved, while radiographic evaluation demonstrated progressive bone healing of the periapical area surrounding the resected implant. Marginal bone levels remained stable, and the emergence profile of the cervical area maintained a satisfactory appearance.

The presented case of microsurgical treatment of retrograde peri-implantitis with a fistula resulted in complete resolution of clinical and radiographic symptoms. CBCT images three months post-implant apex resection revealed healing of the osteolytic defect near the implant apex, with no tendency for progression or recurrence of infection. Twelve-month clinical and radiological follow-up confirmed the complete healing of chronic suppurative peri-implantitis with a fistula. Furthermore, the marginal bone level and soft tissue around the implant remained stable with a normal profile and biological stability. The implant maintained normal function, demonstrated proper secondary stabilization and osseointegration, and had satisfactory esthetics.

## 3. Discussion

With advances in implantology, the number of procedures performed has increased over the years. Individual procedures are becoming more accessible to patients, but at the same time, the rate of complications and failures that practitioners must manage in their daily work is increasing. Microinvasiveness and bone preservation are crucial for the long-term stabilization of prosthetic restorations. Retrograde peri-implantitis (RPI) is a rare but clinically significant disease entity characterized by an inflammatory process involving the apical portion of the implant. At the same time, marginal bone levels and osseointegration remain stable. This clinical and radiographic image distinguishes RPI from marginal peri-implantitis, providing a basis for differentiation and for conservative treatment focused solely on the area limited by the inflammatory process. In recent years, a growing number of reports have indicated that, in appropriately selected cases, RPI can be effectively treated without the need to explant the entire implant, provided secondary stability is maintained and there are no signs of progressive marginal bone loss [9,10,11].

Selected clinical reports describing implant apicoectomy in retrograde peri-implantitis are summarized in Table 1.

From a biological perspective, the removal of a properly integrated implant is associated with significant tissue loss resulting from mechanical damage to the bone walls, disruption of the local vascular network, and an increased risk of unpredictable bone resorption. This is particularly important in the esthetic region of the maxilla, where the thin vestibular bone plate is susceptible to resorption, and secondary implant reconstruction often requires extensive augmentation procedures and is associated with less predictable healing [3,4,5]. In this context, apical resection of the implant eliminates the inflammatory process while simultaneously preserving the hard and soft tissue architecture and the continuity of osseointegration in the coronal portion of the implant. The effectiveness of apical resection in the treatment of RPI stems from its ability to completely remove the implant fragment colonized by bacterial biofilm.

The titanium surface of an implant exhibits high microporosity, which may promote bacterial adhesion and the formation of microbial layers resistant to standard decontamination methods. In such conditions, simply removing the inflammatory tissue and attempting to decontaminate the implant surface, including mechanical, chemical, and laser treatments, may prove insufficient to fully eliminate the source of infection, which can then promote the recurrence of inflammatory lesions [9,14,15,16,17]. Resection of the apical portion of the implant allows for complete removal of the colonized surface. It creates conditions for healing in a biologically “clean” bone, which is evidenced by the observed healing of osteolytic lesions in the case described above [8,9,10,11,18].

Unlike in endodontic surgery, there is no predictable, standardized method for retrograde preparation in implant resection. The implant itself lacks a structure analogous to a root canal, and attempts to apply materials to the cut titanium surface do not replicate its biological or structural properties. In conventional endodontic microsurgery, approximately 3 mm of the tooth root apex is resected to eliminate the apical delta, lateral canals, and most complex apical anatomy, followed by retrograde preparation and root-end filling to seal the root canal system. In contrast, a titanium implant lacks apical ramifications, dentinal tubules, and a complex root canal system; therefore, the inflammatory process associated with retrograde peri-implantitis is not managed by sealing its internal space, but rather by removing the colonized apical implant surface and the surrounding infected tissues. Although guided-regeneration with bone substitute materials and/or membranes has been described as a common method in the surgical management of RPI, regenerative materials were not used in this particular case because the defect was limited, bleeding from surrounding bone was preserved, and primary stabilization of the implant remained unaffected [19]. The surgical objective was to remove the infected apical implant fragment to allow spontaneous bone repair through clot stabilization and natural bone remodeling. Due to previous infections of the same periapical region, it was thought that adding foreign materials could potentially impair bone remodeling and healing.

Current studies emphasize that the key to treatment success is not the reconstruction of the implant surface, but rather the complete removal of the colonized fragment, which, in turn, would promote bone remodeling within the healthy bone [9,11]. From a biomechanical perspective, shortening the implant by a few millimeters does not reduce its stability or function. Careful case selection remains essential when considering implant apicoectomy as an alternative to explantation. Additionally, to preserve marginal bone levels and implant stability, sufficient remaining implant length after apical resection should be considered a critical prerequisite for maintaining favorable biomechanical load distribution and long-term functional stability.

Biomechanical and clinical studies indicate that the coronal and central portions of the implant bear the majority of the occlusal load. In contrast, the apical portion plays a relatively minor role in the distribution of forces acting on the implant [20]. In this context, cutting off the final millimeters of the implant, provided secondary stability and unchanged marginal bone levels are maintained, should not significantly affect its biomechanical behavior. In a study by Balshi et al., a 97.4% survival rate for implants subjected to apical resection at a 4.5-year follow-up was observed, confirming the long-term effectiveness of this procedure [8]. More recent studies also confirm the high effectiveness of implant apex resection in cases of small, limited periapical lesions with preserved implant stability and effective elimination of the infected lesions [10,13,18]. Clinical results from many studies confirm that this procedure is a safe alternative to implant explantation in carefully selected cases.

Another important, yet often overlooked, aspect of choosing a treatment strategy is the economic component. Implant explantation, especially in the esthetic region, typically initiates a multi-stage treatment process involving implant removal, bone regeneration, healing, reimplantation, and prosthetic reconstruction, which results in longer treatment times, increased surgical interventions, and higher financial and biological costs for the patient. Implant apical resection, a limited, targeted procedure, often avoids these stages, thereby shortening treatment time and reducing overall cost.

The presented case aligns with the trend in contemporary implant microsurgery, which emphasizes precise diagnostics and minimally invasive, targeted surgical management. Implant apex resection in the treatment of RPI exemplifies a strategy of removing only the pathological structures while preserving biologically stable tissues, allowing for lasting healing at minimal biological and functional loss [9,10,11]. Despite these favorable clinical characteristics, the differential diagnosis in the presented case requires careful consideration. The exact origin of the inflammatory lesion cannot be determined with absolute certainty, particularly given the history of the chronic periapical pathology and previous apicoectomies associated with tooth 21. Histopathological examination of the tissue removed during implant apicoectomy revealed features consistent with a radicular cyst (cystic radicularis), supporting the presence of pre-existing odontogenic inflammatory pathology. Therefore, the possibility of residual infection in the surrounding tissues cannot be completely ruled out. Nevertheless, the overall clinical and radiological presentation was most consistent with retrograde peri-implantitis. Following immediate implant placement, the initial postoperative healing period was uneventful, with no pain, swelling, or clinical signs of infection. Symptoms only appeared three months later, when a fistula developed in the apical region of the implant. Additionally, the lesion remained confined exclusively to the apical portion of the implant, while marginal bone levels and implant stability were preserved throughout the observation period. The absence of implant mobility, lack of marginal peri-implant bone loss, and maintenance of secondary stability further supported the diagnosis of localized RPI rather than generalized implant failure or persistent postoperative infection. Collectively, these findings suggest that the clinical presentation was most compatible with retrograde peri-implantitis.

Although the clinical outcome in the presented case was favorable, further studies are still necessary to standardize implant apical resection protocols, particularly regarding the optimal extent of apical resection, the selection of regenerative materials, the timing of prosthetic loading, and the histopathological evaluation of tissues in the resected region.

One of the pioneering studies in implant apicoectomy was conducted by McAllister et al. (1992), which described the surgical treatment of implants with periapical radiolucencies and a fistula, aimed at eliminating localized apical pathologies [12]. Subsequent publications, including those conducted by Balshi et al., established the clinical validity of this procedure, although their efficacy assessment was based primarily on clinical and radiological criteria [8]. Histological data on the nature of periapical lesions around implants are rare, as in the study by Luongo et al., which shows granulation tissue with inflammatory infiltration and bacterial colonization limited to the apical part of the implant [13]. Histopathological evaluation of inflammatory tissue removed during implant apex resection can provide information on the nature of the periapical lesion, its degree of progression, and potential mechanisms of development. It should be emphasized, however, that this study examines only pathological tissue removal at the time of surgery and does not allow assessment of the healing process within the resected zone. Healing begins only after the colonized implant fragment is eliminated and the inflammatory lesion is removed. Its course can only be reliably assessed based on clinical and radiological observations in the postoperative period; therefore, routine histopathological examination is not standard in the treatment of retrograde peri-implantitis and is not a prerequisite for assessing the efficacy of the treatment.

Contemporary publications emphasize that the key to success in treating RPI is not surgical aggression, but precise diagnosis and minimal local intervention. Apical resection in this context is an example of targeted minimal surgery—a concept in which only the pathological component is removed, leaving intact what is already biologically stable. Microsurgically, maintaining the integrity of the implant bed supports tissue homeostasis and blood supply, promoting true, not merely radiological, healing. This shift in philosophy from “destructive resection” to “controlled resection” opens a new chapter in the treatment of peri-graft complications—one in which biology becomes the surgeon’s tool, not its victim. This is a new chapter in implant microsurgery, where comprehensive treatment of potential risk factors, including surgical intervention in the case of an implant affected by retrograde periapical inflammation, offers the potential for long-term implant survival [11].

When discussing RPI, it is important to address its causes and prevalence. Sinsareekul reports that the incidence of retrograde peri-implantitis ranged from 0.9% to 23.8% [11]. Higher rates were observed in periapical lesions of extracted or adjacent teeth, periodontitis at the extraction site, and a shorter distance between the implant and adjacent teeth. After a 1-year follow-up, surgical treatment of RPI demonstrated survival rates of 66.7% to 100%, while endodontic treatment of the adjacent tooth yielded success rates of 49% to 100%. The clinical situation described in this manuscript involved immediate implantation after tooth extraction, with a diagnosis of purulent chronic periapical inflammation and a fistula; i.e., it was an inflammatory area. It can be assumed that the non-radical removal of granulation tissue or the presence of vegetative forms of bacteria in the surrounding tissues could have caused RPI.

## 4. Conclusions

Microsurgical implant apicoectomy is a biologically justified treatment for retrograde peri-implantitis if:

1. Osseointegration is maintained clinically and radiographically.
2. Soft tissues and marginal bone are healthy, and the lesion is limited to the apical region of the implant.
3. Sufficient residual implant length remains after apical resection to maintain biomechanical stability.

The treatment used in this clinical situation preserved the function and esthetics of the resected implant at a minimal biological cost. Prospective studies with long-term follow-up (>5 years) are necessary to compare implant apical resection with implant explantation with respect to bone healing, prosthetic stability, and patient satisfaction.

Despite the promising clinical results, the implant apical resection procedure requires further standardization and observation.

## Figures and Tables

**Figure 1:**
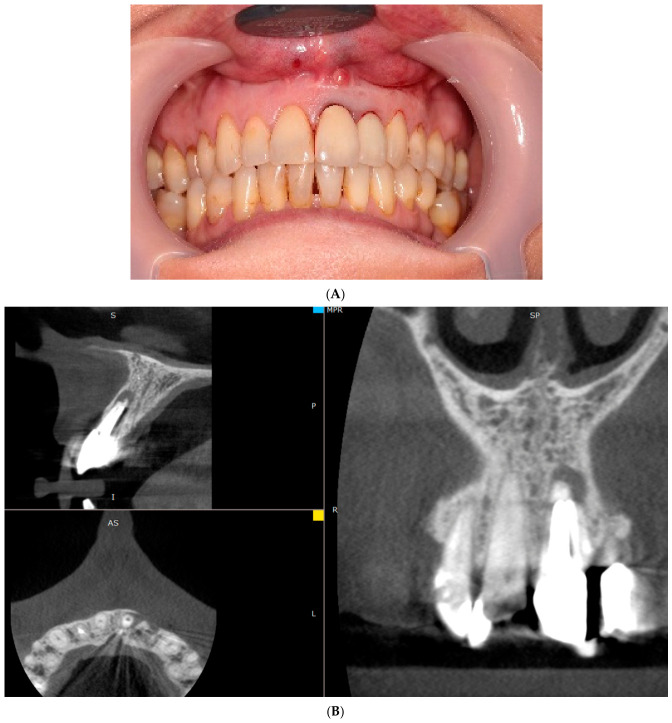
(**A**) Clinical presentation at initial consultation showing a purulent fistula associated with tooth 21; (**B**) Preoperative CBCT showing a chronic inflammatory process at the apex of tooth 21.

**Figure 2:**
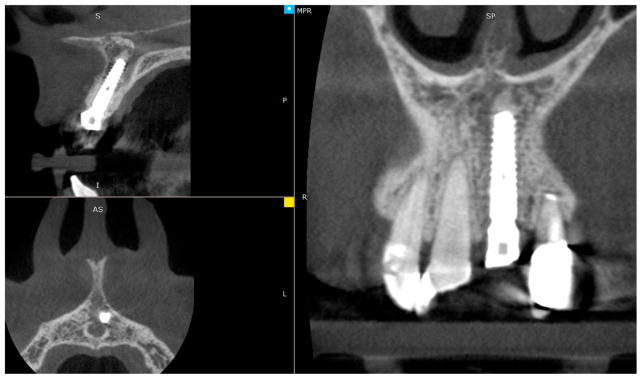
Postoperative CBCT after immediate implant placement of tooth 21.

**Figure 3:**
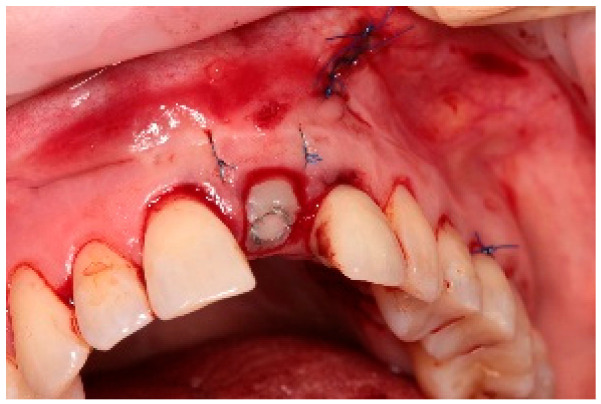
Immediate implant placement with simultaneous bone augmentation, soft tissue enhancement (sCTG), and individualized healing abutment.

**Figure 4:**
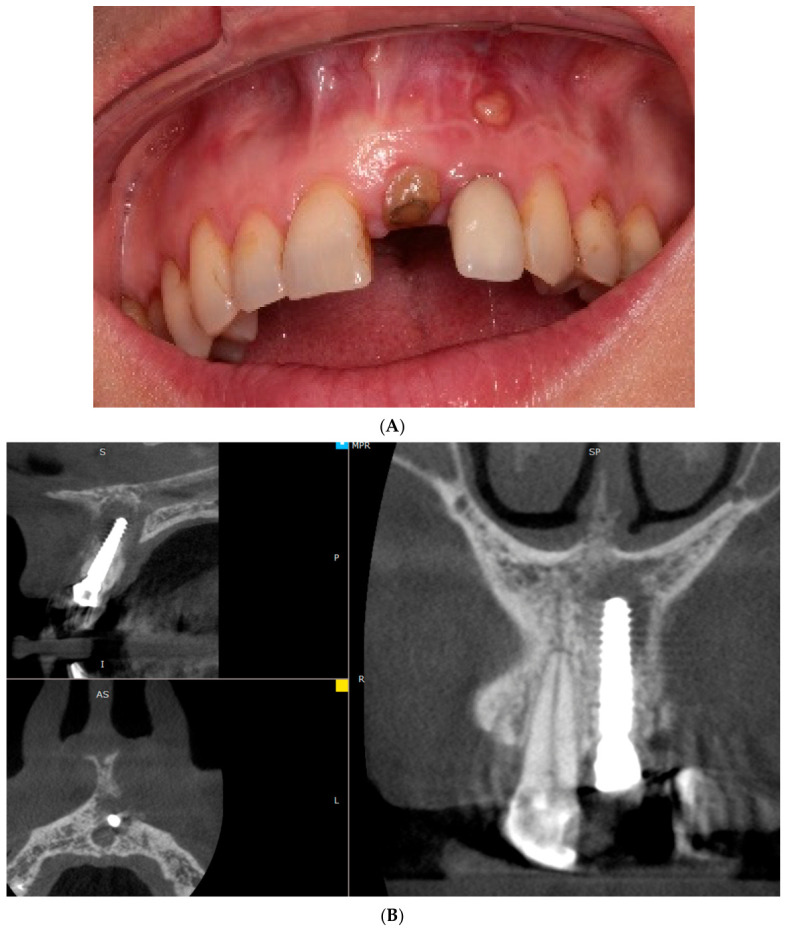
(**A**) Three-month-follow up: persistent fistula corresponding to a chronic inflammatory process at the apical portion of the implant; (**B**) CBCT after three months—periapical lesion limited to the apical part of the implant; marginal bone level remains stable.

**Figure 5:**
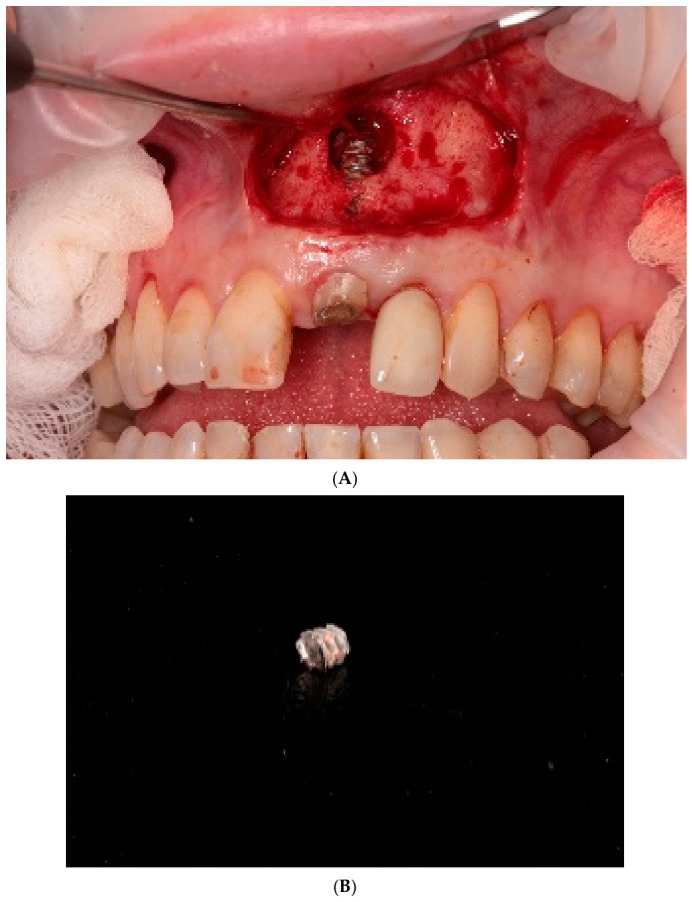

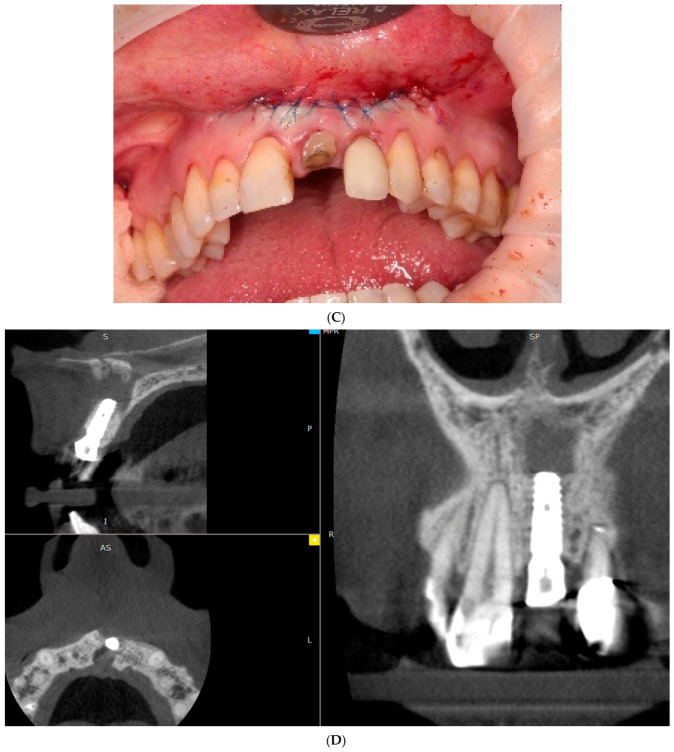
(**A**) Surgical exposure and resection of the apical part of the implant with removal of granulation tissue; (**B**) Resected apical fragment of the implant; (**C**) Wound closure demonstrating tension-free primary healing using monofilament sutures; (**D**) Postoperative CBCT following apical resection of the implant.

**Figure 6:**
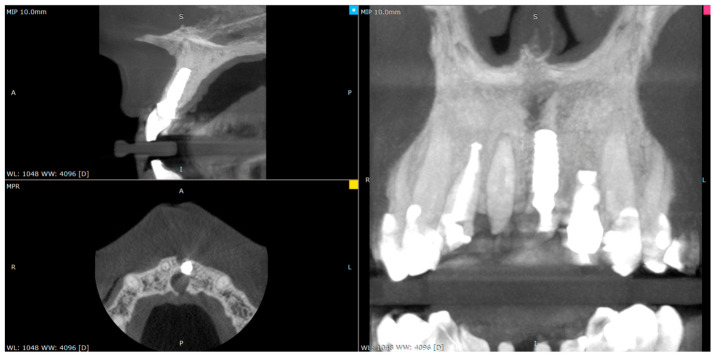
CBCT obtained at follow-up demonstrating marked reduction in the osteolytic lesion and progressive bone healing in the region of the resected implant apex. Marginal bone levels remained stable.

**Figure 7:**
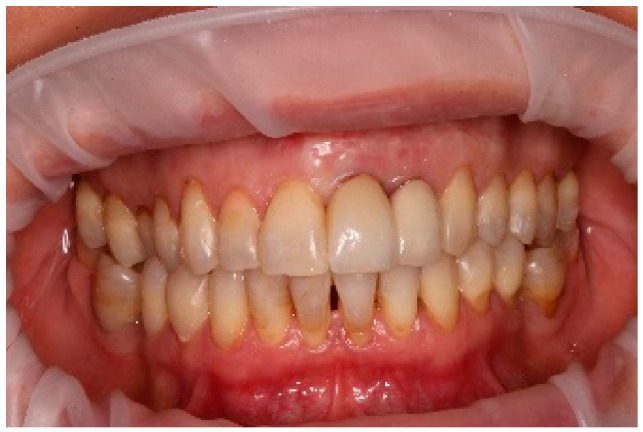
State after healing with final prosthetic reconstruction.

**Table 1:** Selected clinical reports on implant apicoectomy for retrograde peri-implantitis.

Author	Year	Study Design	Number of Implants	Follow-Up Period	Outcome
McAllister BS et al. [12]	1992	Case series	4	12 months	Clinical resolution reported
Balshi SF et al. [8]	2007	Retrospective study	39	Mean 4.5 years	97.4% implant survival
Luongo R et al. [13]	2022	Case series (histological evaluation)	2	12 months	Resolution with histological confirmation
Hoffmann MJ et al. [9]	2022	Case report	1	18 months	Stable implant, no recurrence
Sinsareekul C, Limpuangthip N [11]	2025	Systematic review	—	—	Surgical success rate 66.7–100%

## Data Availability

The original contributions presented in this study are included in the article. Further inquiries can be directed to the corresponding author.

## References

[1] Reiser G.M., Nevins M. (1995). The implant periapical lesion: Etiology, prevention, and treatment. Compend. Contin. Educ. Dent..

[2] Sarmast N.D., Wang H.H., Soldatos N.K., Angelov N., Dorn S.O., Yukna R.A. (2016). A novel treatment decision tree and literature review of retrograde peri-implantitis. J. Periodontol..

[3] Masaki C. (2024). Treatment strategies for dental implant removal: A literature review. Jpn. Dent. Sci. Rev..

[4] Kochar S.P., Reche A., Paul P. (2022). The etiology and management of dental implant failure: A review. Cureus.

[5] Tan W.L., Wong T.L.T., Wong M.C.M., Lang N.P. (2012). A systematic review of post-extractional alveolar hard and soft tissue dimensional changes in humans. Clin. Oral Implant. Res..

[6] Dahlin C., Nikfarid N., Albrektsson T. (2009). Apical peri-implantitis: Possible predisposing factors, case reports, and surgical treatment suggestions. Clin. Implant Dent. Relat. Res..

[7] Boeiriu S., Hottel T.L., Saltz A.E., Chirla C., Chirla P., Hottel S.A., Boeiriu T. (2010). A novel approach for retrograde peri-implantitis treatment using resection and PRGF-Endoret regeneration. J. Periodontol..

[8] Balshi S.F., Wolfinger G.J., Balshi T.J. (2007). A retrospective evaluation of a treatment protocol for dental implant periapical lesions: Long-term results of 39 implant apicoectomies. Int. J. Oral Maxillofac. Implant..

[9] Hoffmann M.J., Hale D.D., Hale K.L. (2022). One alternative surgical treatment for retrograde peri-implantitis: A case report. Front. Dent. Med..

[10] Gao Y., Ma J. (2024). Prevention of retrograde peri-implantitis caused by pulpal/periapical lesions in adjacent teeth: A literature review. J. Dent..

[11] Sinsareekul C., Limpuangthip N. (2025). Potential risks, treatment strategies, and treatment outcomes of retrograde peri-implantitis: A systematic review. J. Prosthet. Dent..

[12] McAllister B.S., Masters D., Meffert R.M. (1992). Treatment of implants demonstrating periapical radiolucencies. Pract. Periodontics Aesthetic Dent..

[13] Luongo R., Sbordone C., Rescigno F., D’Ambrosio M., Sbordone L. (2022). Implant periapical lesion: Clinical and histological findings in two human cases. Int. J. Environ. Res. Public Health.

[14] Dhaliwal J.S., Abd Rahman N.A., Ming L.C., Dhaliwal S.K.S., Knights J., Albuquerque Junior R.F. (2021). Microbial biofilm decontamination on dental implant surfaces: A mini-review. Front. Cell. Infect. Microbiol..

[15] Souza J.G.S., Bertolini M., Zhang Y., Dongari-Bagtzoglou A. (2020). Targeting implant-associated infections: Titanium surface and biofilm formation. Bioengineering.

[16] Silva R.C., Agrelli A., Andrade A.N., Mendes-Marques C.L., Arruda I.R., Santos L.R. (2022). Titanium dental implants: An overview of applied nanobiotechnology to improve biocompatibility and prevent infections. Materials.

[17] Shokeen B., Zamani L., Zadmehr S., Pouraghaie S., Ozawa R., Yilmaz B., Lilak S., Sharma S., Ogawa T., Moshaverinia A. (2021). Surface characterization and assessment of biofilm formation on two titanium-based implant coating materials. Front. Dent. Med..

[18] Koschnitzki E., Surgrue J.P., Weber M.I. (2025). Implant apicoectomy to treat implant-induced chronic sinusitis: A novel approach. J. Oral Implantol..

[19] Banjar A.A. (2025). Retrograde Peri-implantitis: A Scoping Review of Current Understanding of a Rare Complication in Dental Implants. J. Oral Implantol..

[20] Himmlová L., Dostálová T., Kácovský A., Konvickova S. (2004). Influence of implant length and diameter on stress distribution: A finite element analysis. J. Prosthet. Dent..

